# Adjuvant treatment preferences in high-risk upper tract urothelial carcinoma: the perspective of Portuguese medical oncologists

**DOI:** 10.1093/oncolo/oyaf365

**Published:** 2025-10-30

**Authors:** Guilherme Vilhais, Mário Fontes-Sousa

**Affiliations:** Hematology and Oncology Department, CUF Oncology, Lisbon 1350-352, Portugal; Hematology and Oncology Department, CUF Oncology, Lisbon 1350-352, Portugal; Medical Oncology Department, ULS Lisboa Ocidental, Lisbon 1449-005, Portugal

**Keywords:** upper tract urothelial carcinoma, platinum-based chemotherapy, immune checkpoint inhibitors

## Abstract

Although upper tract urothelial carcinoma (UTUC) and bladder urothelial carcinoma (BUC) share histological features, they differ in clinical behavior and management. Valid adjuvant options include surveillance, platinum-based chemotherapy, and immune checkpoint inhibitors (ICIs). To assess real-world practice, we conducted a survey among Portuguese medical oncologists dedicated to genitourinary malignancies, exploring their preferences for adjuvant therapy in high-risk UTUC (illustrated as pT2N1M0) across three clinical scenarios that differed by PD-L1 status and renal function. Among 34 respondents, cisplatin plus gemcitabine was the preferred regimen in cisplatin-eligible patients, regardless of PD-L1 status (94% in PD-L1-negative and 85% in PD-L1-positive tumors). In PD-L1-positive, cisplatin-ineligible patients, carboplatin plus gemcitabine was preferred (47%), followed by ICIs (44%). These findings suggest a consistent preference for platinum-based chemotherapy, likely reflecting UTUC-specific evidence from the POUT trial and apparent limited ICI benefit in this subgroup, underscoring the need for dedicated prospective trials.

## Background

Upper tract urothelial carcinomas (UTUC) are uncommon malignancies of the renal pelvis or ureter, with an estimated incidence of approximately 2 cases per 100 000 individuals annually.^1^ Although UTUC and Bladder Urothelial Carcinoma (BUC) share histological features, they differ significantly in their epidemiological, clinical, pathological, and molecular profiles.^2^^,^^3^ Unlike BUC, where neoadjuvant systemic therapy is the standard of care, neoadjuvant treatment is generally not the preferred approach in UTUC due to lacking prospective data, emphasizing the importance of adjuvant strategies.^4^

Given that 90%-95% of urothelial carcinomas (UC) originate in the bladder, most pivotal clinical trials that granted treatment approvals have included few UTUC patients, and treatment recommendations are frequently extrapolated from BUC evidence.

The POUT trial remains the only phase III randomized study specifically designed for UTUC.^5^ In this trial, 261 patients with high-risk UTUC post-radical nephroureterectomy (RNU) were randomized to receive adjuvant platinum-based chemotherapy (ChT) or surveillance. A statistically significant improvement in disease-free survival (DFS), the primary endpoint, was observed in the ChT arm (Table 1). Importantly, the benefit was fairly consistent across subgroups, including those receiving carboplatin+gemcitabine—a relevant finding, given the reduced cisplatin eligibility post-RNU. Although not adequately powered for Overall Survival (OS) analysis, a positive trend was observed.^6^

**Table 1.** Analysis of clinical trials evaluating adjuvant strategies in high-risk localized UTUC.

Trial	Intervention/Comparator	Population	UTUC %	Primary endpoint(s)	DFS in ITT	DFS in UTUC	OS
**POUT**	Platinum ChT vs. Observation	pT2-pT4 and/or pN+	100	DFS	HR = 0.45, 95% CI 0.30-0.68; *P* = .0001	same as ITT	**Secondary** HR = 0.68; 95% CI: 0.46-1.00; *P* = .049
**IMvigor010**	Atezolizumab vs. Observation	ypT2-T4 and/or ypN+ pT3-T4 and/or pN+	7	DFS	HR = 0.89, 95% CI 0.74-1.08; *P* = .24	HR = 1.25, 95% CI 0.57-2.74	**Secondary** HR 0.91, 95% CI 0.73-1.13
**CheckMate274**	Nivolumab vs. Placebo	ypT2-T4a and/or ypN+ pT3-T4a and/or pN+	21	DFS in ITT population DFS in PD-L1 ≥ 1%	HR = 0.70, 98.22% CI 0.55-0.90; *P* < .001 HR = 0.55, 98.72% CI 0.35-0.85; *P* < .001	**Renal pelvis** HR = 1.23, 95% CI 0.67-2.23 **Ureter** HR = 1.56, 95% CI 0.70-3.48	**Secondary** HR 0.76, 95% CI 0.61-0.96 HR 0.56, 95% CI 0.36-0.86
**AMBASSADOR/KEYNOTE-123**	Pembrolizumab vs. Observation	≥ypT2 and/or ypN+ and/or R1 ≥pT3 or N+ and/or R1	21.9	DFS OS	HR = 0.73, 95% CI 0.59-0.90; *P* = .003	**Renal pelvis** HR = 1.96, 95% CI 0.92-4.17 **Ureter** HR = 1.56, 95% CI 0.77-2.02	**Dual primary** HR = 0.98, 95% CI 0.76-1.26

Abbreviations: ChT, chemotherapy; CI, confidence interval; DFS, disease-free survival; HR, hazard ratio; ITT, intention-to-treat; PD-L1, programmed death ligand 1; OS, overall survival; UTUC, upper tract urothelial carcinoma.

Note: This is not intended as a comparison between trials, but rather as a summary of the available data.

Given the success of immune checkpoint inhibitors (ICIs) in metastatic BUC, their efficacy has also been assessed in the adjuvant setting. The phase III CheckMate 274 trial randomized 709 patients with high-risk UC following radical surgery to receive adjuvant nivolumab or placebo for 1 year.^7^ The trial met its dual primary endpoints, demonstrating a DFS benefit in the intention-to-treat population and in patients with PD-L1 expression ≥1%. Nivolumab was subsequently approved in this setting by the European Medicines Agency for PD-L1 positive tumors and the U.S. Food and Drug Administration, regardless of PD-L1 status. UTUC patients comprised 21% of the study population—higher than typically observed—but subgroup analysis suggested limited benefit compared to BUC (Table 1).

The AMBASSADOR (KEYNOTE-123) trial randomized 739 patients with high-risk UC to receive adjuvant pembrolizumab for 1 year or observation.^8^ DFS was improved in the pembrolizumab arm, independently of PD-L1 expression. Final OS data are pending, but preliminary analyses have not shown a clear benefit. Of note, the approval of nivolumab during trial enrollment may have led to treatment crossover, potentially confounding OS interpretation. This trial also included 21.9% UTUC patients, and, as with CheckMate 274, subgroup analysis did not indicate a significant benefit in this subgroup (Table 1).

Atezolizumab was also evaluated in this setting, but the trial did not meet its primary endpoint of improved DFS (Table 1).^9^

Current evidence highlights the uncertainty surrounding the optimal adjuvant approach in high-risk localized UTUC, which accounts for approximately 40%-50% of all patients with UTUC.^10^ Platinum-based ChT demonstrated a DFS benefit (and OS trend) in a UTUC-specific trial, whereas ICIs showed a DFS benefit (and OS with nivolumab) in broader UC populations that appears to be driven primarily by BUC. The reasons behind the apparently reduced benefit of ICIs in UTUC remain unclear, despite the higher prevalence of Lynch syndrome and microsatellite instability-high (MSI-H) tumors in UTUC, which are generally predictive of ICI responsiveness.

## Real-world survey results and discussion

In order to explore current clinical practice preferences, we conducted a national real-world anonymous survey (Supplement 1) among Portuguese medical oncologists dedicated to genitourinary malignancies. Of 50 personally invited oncologists, 34 responded (68%), with balanced geographic representation and predominant practice in public institutions. Most respondents (67.6%) had been practicing medical oncology for fewer than 10 years, likely reflecting the national oncology workforce; however, potential selection bias cannot be excluded. Regarding UTUC caseload, 41.2% reported treating more than 5 patients with localized UTUC annually, 44.1% reported treating 3-5, and 14.7% fewer than 3, representing a substantial proportion of the national UTUC incidence (Table S1).

As neoadjuvant chemotherapy (NAC) may influence subsequent adjuvant strategies, respondents were first asked about its role. A majority (58.8%) considered NAC appropriate in selected cases, while 32.4% reported NAC as their preferred approach, likely reflecting concerns regarding post-RNU cisplatin ineligibility. A minority (8.8%) never considers NAC appropriate, possibly due to the lack of prospective data.

**Figure 1.**
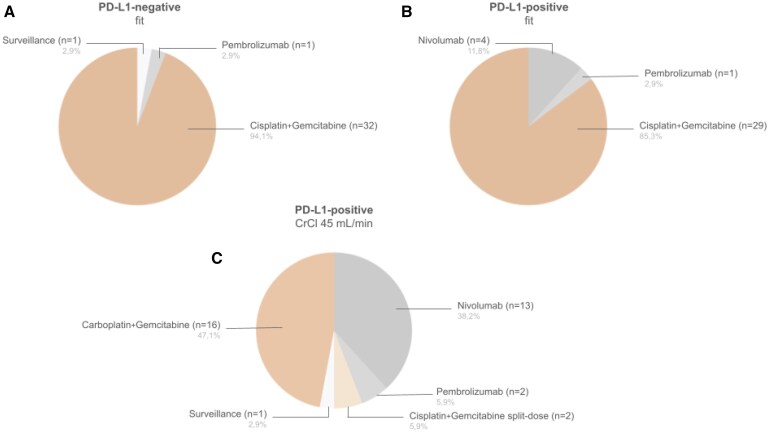
Selected adjuvant strategies in high-risk localized UTUC for each clinical scenario by Portuguese medical oncologists that responded to the real-world survey. CrCl, creatinine clearance; PD-L1, programmed death ligand 1.

Three clinical scenarios were presented, all involving high-risk UTUC (illustrated as pT2pN1M0) R0, of the renal pelvis post-RNU, and therefore eligible for POUT, CheckMate 274, and AMBASSADOR trials (Figure 1). They were then asked to select the most appropriate adjuvant strategy based on available evidence, irrespective of regulatory approvals. The scenarios varied by PD-L1 status and renal function:

- **Case 1**: PD-L1 negative, fit (no comorbidities)
- **Case 2**: PD-L1 positive, fit (no comorbidities)
- **Case 3**: PD-L1 positive, creatinine clearance (CrCl) 45 mL/min

Treatment options included surveillance, cisplatin+gemcitabine (with split-dose for case 3), carboplatin+gemcitabine, nivolumab and pembrolizumab.

In case 1, 94.1% selected cisplatin+gemcitabine as their preferred adjuvant choice, decreasing to 85.3% in case 2 (Figure 1A and B). In case 3, carboplatin+gemcitabine was slightly preferred (47.1%) over ICIs (44.1%), with only 5.9% choosing split-dose cisplatin (Figure 1C). These results suggest that platinum-based ChT remains the preferred adjuvant treatment, even in PD-L1-positive patients. In cisplatin-ineligible patients (CrCl < 60 mL/min),^11^ carboplatin+gemcitabine was slightly favored over ICIs, possibly reflecting awareness of the POUT subgroup data and the inconclusive benefit of ICIs in UTUC-specific subgroups. We attribute the low preference for split-dose cisplatin to the fact that it was not permitted in the POUT trial. This strategy was recently validated in UC in the NIAGARA trial (CrCl ≥ 40 mL/min); however, the trial did not include UTUC patients.^12^

## Future directions

This survey highlights that respondent physicians tend to prioritize evidence from trials conducted specifically in UTUC, with platinum-based ChT remaining the mainstay adjuvant therapy, as recommended by guidelines.^4^ Choice of ICIs appears to be predominantly considered for PD-L1-positive cisplatin-ineligible patients.

Importantly, 97.1% of respondents indicated willingness to enroll patients in a randomized trial comparing adjuvant ChT to ICI monotherapy in high-risk localized UTUC. Such a trial could address whether UTUC are intrinsically less responsive to ICIs, and help identify subgroups (eg, MSI-H or Lynch syndrome) more likely to benefit. Future studies could also help define future lines of research/clinical trial development, namely with newer treatment combination regimens (eg, ICI+Antibody Drug Conjugate), as well as in selecting the most appropriate comparators and potentially identifying biomarkers for treatment selection.

## Supplementary Material

oyaf365_Supplementary_Data

## Data Availability

The data underlying this article will be shared on reasonable request to the corresponding author.
