# Supplementary material for: Adjuvant treatment preferences in high-risk upper tract urothelial carcinoma: the perspective of Portuguese medical oncologists
Source: Oncologist. 2025 Oct 30;30(11):oyaf365. doi: 10.1093/oncolo/oyaf365 (PMC12619994; doi:10.1093/oncolo/oyaf365)
Supplement: oyaf365_Supplementary_Data [file oyaf365_supplementary_data.zip › Supplementary Material - Table S1.docx]

|  | **n (%)** |
| --- | --- |
| **Geographical region** |  |
| North | 10 (29.4) |
| Center | 10 (29.4) |
| South and Islands | 14 (41.2) |
| **Practice setting** |  |
| Exclusively public | 19 (55.9) |
| Exclusively private | 8 (23.5) |
| Mixed | 7 (20.6) |
| **Practice time as specialist** |  |
| < 10 years | 23 (67.6) |
| ≥ 10 years | 11 (32.4) |
| **Average number of localized UTUC patients treated/year** |  |
| < 3 | 5 (14.7) |
| 3 to 5 | 15 (44.1) |
| > 5 | 14 (41.2) |

**Table S1** - Characterization of Portuguese medical oncologists that responded to the real-world survey.
