# Supplementary material for: Adjuvant treatment preferences in high-risk upper tract urothelial carcinoma: the perspective of Portuguese medical oncologists
Source: Oncologist. 2025 Oct 30;30(11):oyaf365. doi: 10.1093/oncolo/oyaf365 (PMC12619994; doi:10.1093/oncolo/oyaf365)
Supplement: oyaf365_Supplementary_Data [file oyaf365_supplementary_data.zip › Supplementary Material - Questionnaire.docx]

**Supplementary Material**

**Questionnaire – (Neo)Adjuvant Treatment in UTUC in Portugal**

**1. Where do you currently practice?** (Select only one option)

- North
- Center
- South and Islands

**2. Type of clinical practice?** (Mark all that apply)

- Public – Central Hospital
- Public – Portuguese Institute of Oncology
- Public – District Hospital
- Private

**3. Years of experience as a specialist in Oncology?** (Select only one option)

- < 10 years
- ≥ 10 years

**4. On average, how many patients with early-stage UTUC do you treat per year?** (Select only one option)

- < 3
- 3 to 5
- > 5

**5. Regarding the role of neoadjuvant treatment in UTUC in your clinical practice:** (Select only one option)

- I consider it the preferred option in these cases
- I consider it a valid option in selected cases
- I believe it has no role

**6. Consider the following clinical case:**

**Patient diagnosed with renal pelvis urothelial carcinoma, pT2pN1M0, R0, PDL1-negative, with no comorbidities preventing any treatment option.**

**Based on the available evidence, regardless of regulatory approvals, which postoperative approach would you consider most appropriate for this patient?** (Select only one option)

- Surveillance
- Cisplatin/Gemcitabine (as per POUT)
- Carboplatin/Gemcitabine (as per POUT)
- Nivolumab (as per CheckMate274)
- Pembrolizumab (as per AMBASSADOR/Keynote123)

**7. Consider the same clinical case, but the patient is PDL1-positive.**

**Based on the available evidence, regardless of regulatory approvals, which postoperative approach would you consider most appropriate?** (Select only one option)

- Surveillance
- Cisplatin/Gemcitabine (as per POUT)
- Carboplatin/Gemcitabine (as per POUT)
- Nivolumab (as per CheckMate274)
- Pembrolizumab (as per AMBASSADOR/Keynote123)

**8. Now consider the same case (PDL1-positive), but the patient has a creatinine clearance of 45 mL/min.**

**Based on the available evidence, regardless of regulatory approvals, which postoperative approach would you consider most appropriate?** (Select only one option)

- Surveillance
- Split-dose Cisplatin/Gemcitabine
- Carboplatin/Gemcitabine (as per POUT)
- Nivolumab (as per CheckMate274)
- Pembrolizumab (as per AMBASSADOR/Keynote123)

**9. If there were a hypothetical phase IV clinical trial randomizing high-risk UTUC patients (≥pT3 and/or pN+ and/or positive margins) between adjuvant platinum-based chemotherapy (as per POUT) or adjuvant immune checkpoint inhibitors (Nivolumab or Pembrolizumab, depending on PDL1 expression), would you consider enrolling patients in this trial?** (Select only one option)

- Yes
- No
